# Longitudinal patient-reported outcomes after minimally invasive McKeown esophagectomy for patients with esophageal squamous cell carcinoma

**DOI:** 10.1007/s00520-024-08428-z

**Published:** 2024-03-20

**Authors:** Yan Miao, Xin Nie, Wen-Wu He, Chun-Yan Luo, Yan Xia, Ao-Ru Zhou, Si-Rui Wei, Cheng-Hao Wang, Qiang Fang, Lin Peng, Xue-Feng Leng, Yong-Tao Han, Lei Luo, Qin Xie

**Affiliations:** Department of Thoracic Surgery, Sichuan Cancer Research Center for Cancer, Sichuan Cancer Hospital and Institute, Sichuan Cancer Center, Affiliated Cancer Hospital of University of Electronic Science and Technology of China, Chengdu, Sichuan 610041 People’s Republic of China

**Keywords:** Esophageal squamous cell carcinoma, McKeown esophagectomy, Patient-reported outcomes, Quality of life

## Abstract

**Purpose:**

Surgery for esophageal squamous cell carcinoma (ESCC) is characterized by a poor prognosis and high complication rate, resulting in a heavy symptom burden and poor health-related quality of life (QOL). We evaluated longitudinal patient-reported outcomes (PROs) to analyze the correlations between symptoms and QOL and their changing characteristics during postoperative rehabilitation.

**Methods:**

We investigated patients with ESCC who underwent minimally invasive McKeown esophagectomy at Sichuan Cancer Hospital between April 2019 and December 2019. Longitudinal data of the clinical characteristics and PROs were collected. The MD Anderson Symptom Inventory and European Organization for Research and Treatment of Cancer (EORTC) QOL questionnaires were used to assess symptoms and QOL and compare the trajectories of PROs during the investigation.

**Results:**

A total of 244 patients with ESCC were enrolled in this study. Regarding QOL, role and emotional functions returned to baseline at 1 month after surgery, and cognitive and social functions returned to baseline at 3 months after surgery. However, physical function and global QOL did not return to baseline at 1 year after surgery. At 7 days and 1, 3, 6, and 12 months after surgery, the main symptoms of the patients were negatively correlated with physical, role, emotional, cognitive, and social functions and the overall health status (*P* < 0.05).

**Conclusion:**

Patients with ESCC experience reduced health-related QOL and persisting symptoms after minimally invasive McKeown esophagectomy, but a recovery trend was observed within 1 month. The long-term QOL after esophagectomy is acceptable.

## Introduction

Esophageal cancer (EC) is the seventh most common malignant tumor worldwide and has the sixth highest mortality [1–3]. Esophageal squamous cell carcinoma (ESCC) is the primary subtype of EC, especially in China, where it accounts for more than 90% of all cases [4]. Currently, EC is treated using a multidisciplinary treatment model combining surgery, radiotherapy, and chemotherapy [5, 6]. The choice of individualized therapy primarily depends on the disease’s developmental stage, the tumor’s location, and the patient’s general condition. Radical esophagectomy is recommended for all patients with resectable EC, except those with EC confined to the submucosa [5].

The surgical management of EC is characterized by a high incidence of complications, ranging from pulmonary complications to anastomotic fistulas. Of all the surgical treatments for patients with EC, as many as 50% result in severe postoperative complications within 30 days after surgery [7, 8], leading to poor postoperative health-related quality of life (HRQL). After surgery, patients experience clinically relevant long-term deterioration of HRQL [9–11]. Long-term survivors’ quality of life (QOL) can be restored to the preoperative level within 9 months [12]. However, some patients do not fully recover their QOL at 6 months to 5 years after surgery, and the symptoms persist [13]. Therefore, effective perioperative strategies are needed to improve long-term HRQL. Previous studies have shown that minimally invasive esophagectomy results in a low incidence of pulmonary infection 2 weeks after surgery and during hospitalization, short hospitalization time, good short-term QOL, and no reduction in the quality of resected specimens, thus enhancing the recovery of postoperative HRQL [14].

According to the modern medical model, controlling the postoperative symptom burden and improving patients’ QOL while prolonging their overall survival (OS) is necessary. In the past 10 years, patient-reported outcomes (PROs) have become increasingly recognized during clinical outcome evaluations and by the United States Food and Drug Administration during drug label declaration trials [15–17]. Many different PRO measures are available for the multidimensional assessment of HRQL [18].

Previously, we studied the signs and QOL of patients at a single time point after surgery; however, greater continuity and long-term follow-up are needed. Hence, during this study, we aimed to provide a sufficient basis for improving the QOL of patients with ESCC after esophagectomy by analyzing the correlation between symptoms and QOL and the changing characteristics of patients with ESCC during postoperative rehabilitation.

## Methods

### Patients

**Fig. 1:**
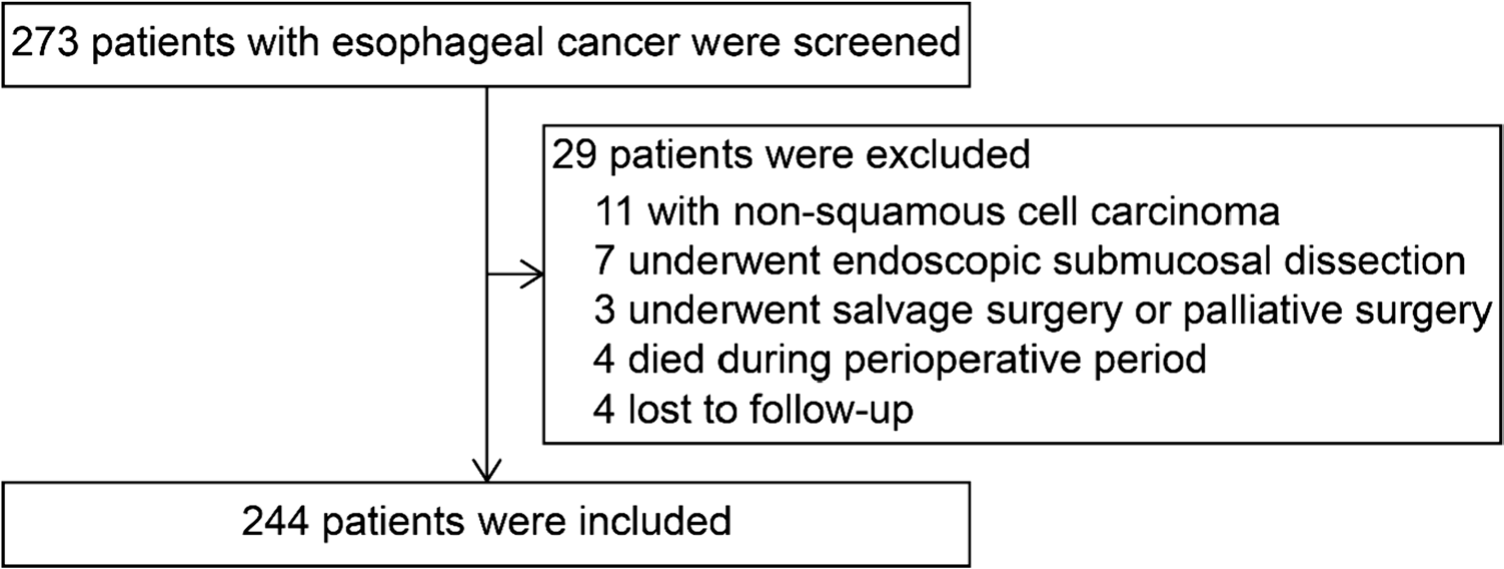
CONSORT diagram of patient selection

From April to December 2019, patients with pathologically confirmed ESCC who underwent potentially curative esophagectomy at the Department of Thoracic Surgery of Sichuan Cancer Hospital were selected as study participants. The inclusion criteria were as follows: (1) age 18 to 80 years; (2) pathological diagnosis of ESCC; (3) underwent minimally invasive McKeown esophagectomy; (4) capability of daily communication and specific understanding ability; and (5) provided informed consent to participate in this study and willingness to cooperate with follow-up. The exclusion criteria were as follows: (1) patients with other tumors; (2) patients with serious physical or mental diseases; and (3) patients who were unaware of their disease. Based on these criteria, 244 patients were included in this study (Fig. 1). This study was performed in line with the principles of the Declaration of Helsinki. Approval was granted by the Ethics Committee (EC) for Medical Research and New Medical Technology of Sichuan Cancer Hospital (approval no: SCCHEC-02–2022-050). All participants provided written informed consent.

### Symptom measurements

The MD Anderson Symptom Inventory [19, 20] was developed in 2000 by the MD Anderson Cancer Center in Texas, USA. It is concise, includes questions regarding the core set of symptoms experienced by patients with cancer, and is easy to understand and complete. The questionnaire was divided into two parts. The first part assessed the symptoms of patients with cancer, including pain, fatigue, nausea, and disturbed sleep. The second part evaluated the extent to which general activity, mood, work, relationships with other people, walking, and enjoyment of life affected daily life using a scale of 0 to 10, with 0 being asymptomatic and 10 being the most severe imaginable. According to the cutoff values, 0 was defined as “no symptoms,” 1–3 as “mild symptoms,” 4–7 as “moderate symptoms,” and 8–10 as “severe symptoms” [21, 22]. In addition, non-zero scores indicate the occurrence of symptoms. The average score of all items in each subscale was used to assess symptom distress. The higher the total score in each section, the more severe the symptoms and the more significant the disruption of life.

### HRQL measurements

HRQL was measured using the European Organization for Research and Treatment of Cancer (EORTC) measure [23]. The EORTCQLQ-C30 is a reliable and effective measure of QOL for patients with cancer in multicultural clinical research settings. The Chinese version of the EORTCQLQ-OES18 is a reliable, effective, and acceptable tool for measuring HRQL of patients with ESCC in mainland China [24]. The instrument has a total of 30 entries divided into the following 15 dimensions: five functional dimensions (physical, role, cognitive, emotional, and social functions); three symptom dimensions (fatigue, pain, and nausea and vomiting); one general health/QOL dimension; and six single entries (each as a dimension). The raw scores for each dimension were calculated using a formula [23, 24], followed by the application of the polarization method for the linear transformation of the raw scores into standard scores. Subsequently, the measurements were compared.

### Data collection

Data collection for this study was completed independently by the researchers, who interpreted the questionnaire items using unified guidelines during the survey to determine the patients with ESCC who met the inclusion criteria. After obtaining informed consent from the patients, the researchers collected general data by consulting with the patients and examining their medical records during hospitalization. Regarding the pulmonary function test, the severity level of airflow obstruction used the percentage of predicted forced expiratory volume in 1 s (FEV1), using cutoff values of 70%, 60%, 50%, and 35%. Combining lung function tests and medical history for lung function diagnosis is necessary. In this study, we used a three-level classification method based on the percentage of normal expected values for ventilation function: mild damage: 60% ≤ FEV1 or diffusing lung capacity for carbon monoxide (DLCO) (or DLCO/VA)% pred < 80%; moderate damage: 40% ≤ FEV1 or DLCO (or DLCO/VA)% pred < 60%; severe damage: FEV1 or DLCO (or DLCO/VA)% pred < 40%. Symptoms and QOL were assessed at enrollment (preoperative baseline) and within 1 year after surgery. Symptom survey time points were 1, 3, 5, 7, 14, and 21 days and 1, 3, 6, and 12 months after surgery. The QOL was surveyed 7 days and 1, 3, 6, and 12 months after surgery. The paper version of the questionnaire was completed during hospitalization, and the assessment was conducted via telephone during follow-up after discharge.

### Statistical analysis

All statistical analyses were performed using SPSS (version 26.0; SPSS Inc., Chicago, IL, USA). Descriptive statistical methods were adopted for the survey participants’ general information, symptoms, and disturbances. The normally distributed data are represented as means ± standard deviations, whereas the non-normally distributed data are represented as medians and interquartile ranges (IQRs). Categorical variables are presented as numbers, percentages, or proportions. Pearson’s correlation analysis was used to determine the relationship between the symptoms and QOL. Linear mixed-effect models were used to evaluate whether symptoms and QOL significantly differed over time. Maximum likelihood estimation was used. A two-sided *p*-value < 0.05 was considered statistically significant.

## Results

### Patient characteristics

The clinicopathological features of 244 patients are shown in Table 1. There were 22 (9.0%), 99 (40.6%), 89 (36.5), and 34 (13.9%) patients with clinical stages I, II, III, and IV, respectively. Seventy-nine (32.4%) patients received neoadjuvant therapy. Among the patients who received neoadjuvant therapy, 43 (54.4%), 9 (11.4%), 24 (30.4%), and 3 (3.8%) patients had post-neoadjuvant pathological stages (ypTNM) I, II, III, and IV, respectively. Additionally, 43 (26.1%), 59 (35.8%), 54 (32.7%), and 9 (5.4%) patients with pathological stages (pTNM) I, II, III, and IV, respectively, underwent surgery without neoadjuvant therapy.

**Table 1:** Characteristics of 244 patients included in the analysis

Variables	No. (%)
Median age (range), year	62.5 (41–84)
Sex	
Male	202 (82.8)
Female	42 (17.2)
Tumor location	
Upper	28 (11.5)
Middle	144 (59.0)
Lower	72 (29.5)
cT stage	
T1	22 (9.0)
T2	35 (14.4)
T3	153 (62.7)
T4	34 (13.9)
cN stage	
N0	105 (43.0)
N +	139 (57.0)
cTNM stage	
I	22 (9.0)
II	99 (40.6)
III	89 (36.5)
IV	34 (13.9)
pTNM stage	
I	43 (26.1)
II	59 (35.8)
III	54 (32.7)
IV	9 (5.4)
ypTNM stage	
I	43 (54.4)
II	9 (11.4)
III	24 (30.4)
IV	3 (3.8)
ECOG score	
1	166 (68)
2	76 (31.2)
3	2 (0.8)
Basic diseases	
Hypertension/diabetes/coronary heart disease	57 (23.4)
None	187 (77.6)
Pulmonary function	
Normal	127 (52.0)
Mild damage	99 (40.6)
Moderate damage	15 (6.2)
Severe damage	3 (1.2)
Neoadjuvant therapy	
Yes	79 (32.4)
No	165 (67.6)
Postoperative adjuvant therapy^a^	
Yes	68 (29.1)
No	166 (70.9)

*ECOG* Eastern Cooperative Oncology Group

^a^*N* = 234 because 10 cases were missing

### Perioperative morbidity

The incidence rates of anastomotic leakage, pneumonia, recurrent laryngeal nerve paresis, chylothorax, pleural effusion, pneumothorax, and arrhythmia in 244 patients after esophagectomy were 15.2%, 20.1%, 9.8%, 2.9%, 25.0%, 3.7%, and 12.7%, respectively (Table 2).

**Table 2:** Perioperative morbidity

Complications	No. (%)
Serious complications (i.e., any events below occurred)	99 (40.6)
Anastomotic leakage	37 (15.2)
Pneumonia	49 (20.1)
Recurrent laryngeal nerve paresis	24 (9.8)
Chylothorax	7 (2.9)
Pleural effusion	61 (25.0)
Pneumothorax	9 (3.7)
Arrhythmia	31 (12.7)

### Incidence of preoperative and postoperative symptoms

**Fig. 2:**
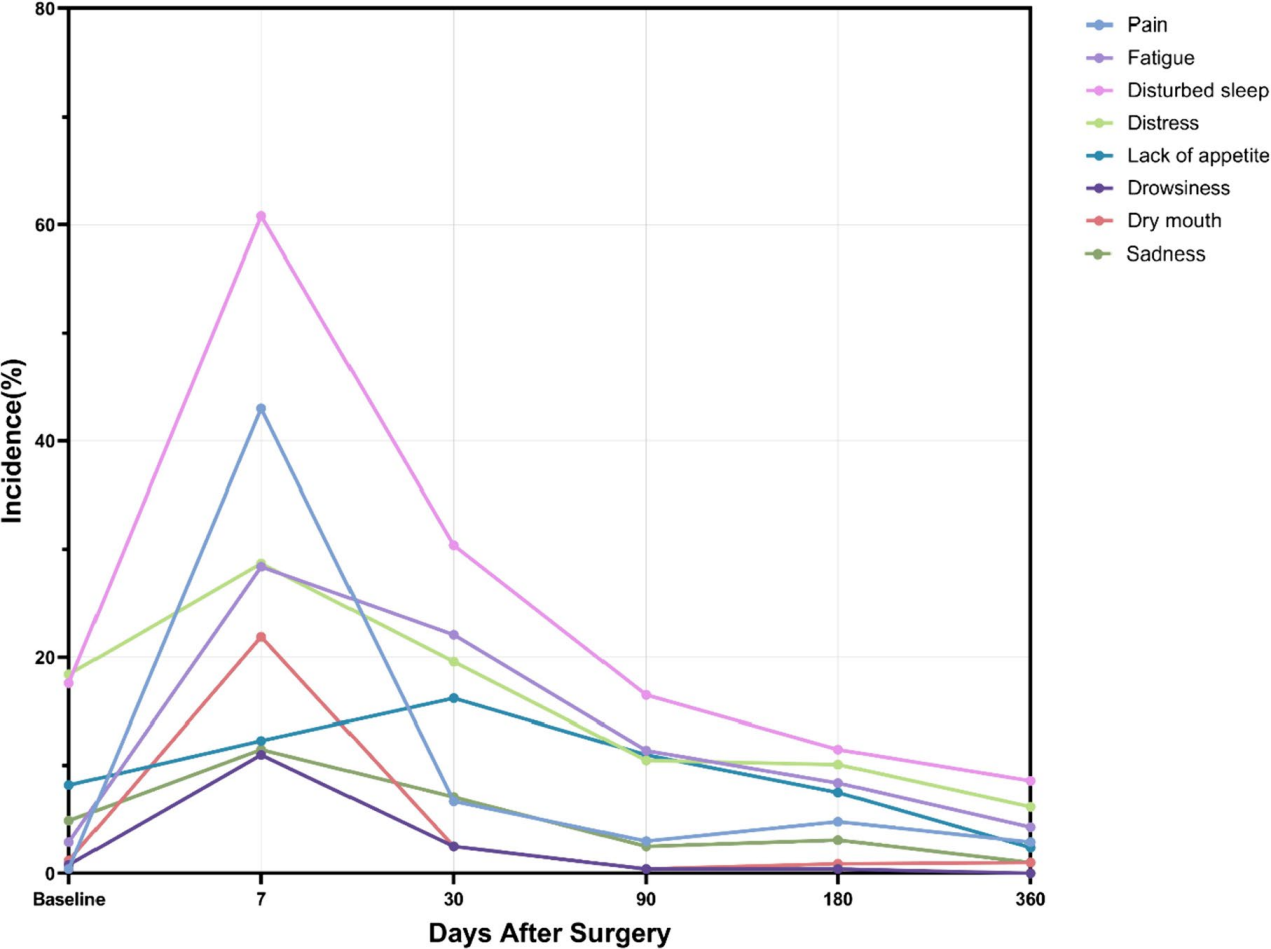
Trends in the incidence of the postoperative burden of moderate to severe symptoms

The incidence and changing trends of pre- and postoperative symptoms of patients with ESCC who underwent minimally invasive McKeown esophagectomy are shown in Table 3. The incidence of all symptoms was more than 80% within 14 days of surgery (symptom score > 0). The incidence of moderate to severe symptoms (symptom score ≥ 4 on a 0–10 scale) peaked shortly after surgery, followed by a significant decrease (Fig. 2).

**Table 3:** Incidence of perioperative symptoms

	Baseline	Postoperative day 1	Postoperative day 3	Postoperative day 5	Postoperative day 7	Postoperative day 14	Postoperative day 21	Postoperative month 1	Postoperative month 3	Postoperative month 6	Postoperative month 12
Pain	26.6	100	99.6	99.1	99.2	96.2	97.1	87.1	61.2	43.2	26.3
Fatigue	35.7	97.4	98.3	95.7	92.8	96.7	95.4	90.4	81	65.2	36.8
Disturbed sleep	55.3	98.3	95.3	96.6	94.5	92.1	83.4	82.9	52.7	34.8	29.2
Distress	93.9	97	98.3	98.7	92.4	98.7	97.5	93.3	78.1	66.1	50.7
Lack of appetite	49.6	87.9	84.6	82.5	85.1	87.5	91.7	89.2	67.1	34.6	11.5
Drowsiness	33.6	99.1	98.7	98.3	87.3	84.2	62.7	49.2	14.8	9.7	1.4
Dry mouth	26.2	97	94	89.3	82.3	57.1	43.2	31.2	4.2	8.8	1.9
Sadness	81.5	94	93.6	96.2	91.9	85	75.1	59.2	27	19.4	5.3

All values are percentage

### Correlation between symptoms and QOL

Before surgery, there was no apparent correlation between patients’ symptoms and QOL. At 7 days, 1 month, 3 months, 6 months, and 12 months after surgery, the main symptoms of the patients were negatively correlated with physical function, role performance, emotional function, cognitive function, social function, and global QOL (*P* < 0.05). Fatigue and distress were moderately correlated with physical function, emotional function, and global QOL (*r* = 0.510–0.785; *P* < 0.01) (Table 4).

**Table 4:** Correlation between symptoms and QOL before and after esophagectomy (*R*-value)

Items	Time	Pain	Fatigue	Disturbed sleep	Distress	Drowsiness	Dry mouth
Physical function	T1	− .113	− .452^**^	− .293^**^	− .190^**^	− .114	− .098
	T2	− .331^**^	− .546^**^	− .363^**^	− .538^**^	− .387^**^	− .391^**^
	T3	− .290^**^	− .642^**^	− .452^**^	− .629^**^	− .390^**^	− .473^**^
	T4	− .412^**^	− .682^**^	− .528^**^	− .621^**^	− .322^**^	− .232^**^
	T5	− .273^**^	− .707^**^	− .473^**^	− .640^**^	− .266^**^	− .013
	T6	− .238^**^	− .578^**^	− .301^**^	− .510^**^	− .146^*^	− .009
Role function	T1	.005	− .024	− .111	− .154^*^	− .165^**^	− .080
	T2	− .178^**^	− .441^**^	− .164^*^	− .385^**^	− .478^**^	− .188^**^
	T3	− .225^**^	− .550^**^	− .471^**^	− .655^**^	− .453^**^	− .531^**^
	T4	− .315^**^	− .463^**^	− .317^**^	− .473^**^	− .413^**^	− .399^**^
	T5	− .213^**^	− .441^**^	− .392^**^	− .449^**^	− .478^**^	− .284^**^
	T6	− .185^**^	− .378^**^	− .259^**^	− .367^**^	− .344^**^	− .137^*^
Emotional function	T1	− .193^**^	− .178^**^	− .179^**^	− .488^**^	− .097	.036
	T2	− .264^**^	− .243^**^	− .244^**^	− .489^**^	− .258^**^	− .235^**^
	T3	− .317^**^	− .488^**^	− .448^**^	− .735^**^	− .350^**^	− .535^**^
	T4	− .418^**^	− .587^**^	− .532^**^	− .713^**^	− .309^**^	− .230^**^
	T5	− .385^**^	− .676^**^	− .461^**^	− .785^**^	− .234^**^	− .151^*^
	T6	− .446^**^	− .516^**^	− .333^**^	− .722^**^	− .135	− .094
Cognitive function	T1	− .199^**^	.001	− .083	− .209^**^	− .029	− .018
	T2	− .212^**^	− .308^**^	− .160^*^	− .387^**^	− .107	− .188^**^
	T3	− .283^**^	− .449^**^	− .381^**^	− .534^**^	− .368^**^	− .349^**^
	T4	− .309^**^	− .360^**^	− .347^**^	− .358^**^	− .297^**^	− .371^**^
	T5	− .128	− .381^**^	− .417^**^	− .358^**^	− .306^**^	− .170^*^
	T6	− .149^*^	− .280^**^	− .330^**^	− .163^*^	− .258^**^	− .104
Social function	T1	.006	− .020	− .075	.035	− .077	− .046
	T2	− .104	− .087	− .110	− .095	− .144^*^	.052
	T3	− .211^**^	− .353^**^	− .175^**^	− .385^**^	− .053	− .235^**^
	T4	− .342^**^	− .445^**^	− .305^**^	− .466^**^	− .373^**^	− .280^**^
	T5	− .250^**^	− .236^**^	− .289^**^	− .324^**^	− .238^**^	− .388^**^
	T6	− .194^**^	− .377^**^	− .257^**^	− .315^**^	− .460^**^	− .251^**^
Global quality of life	T1	− .124	− .342^**^	− .239^**^	− .305^**^	− .116	− .115
	T2	− .389^**^	− .598^**^	− .447^**^	− .661^**^	− .313^**^	− .275^**^
	T3	− .246^**^	− .499^**^	− .354^**^	− .628^**^	− .255^**^	− .444^**^
	T4	− .357^**^	− .649^**^	− .472^**^	− .664^**^	− .334^**^	− .259^**^
	T5	− .389^**^	− .598^**^	− .447^**^	− .661^**^	− .313^**^	− .275^**^
	T6	− .312^**^	− .471^**^	− .317^**^	− .580^**^	− .173^*^	− .192^**^

*QOL* quality of life, *T1* baseline (before surgery), *T2* postoperative day 7, *T3* postoperative month 1, *T4* postoperative month 3, *T5* postoperative month 6, *T6* postoperative month 12

^*^P < 0.05, ^**^P < 0.01

### Longitudinal changes in symptoms and QOL

**Fig. 3:**
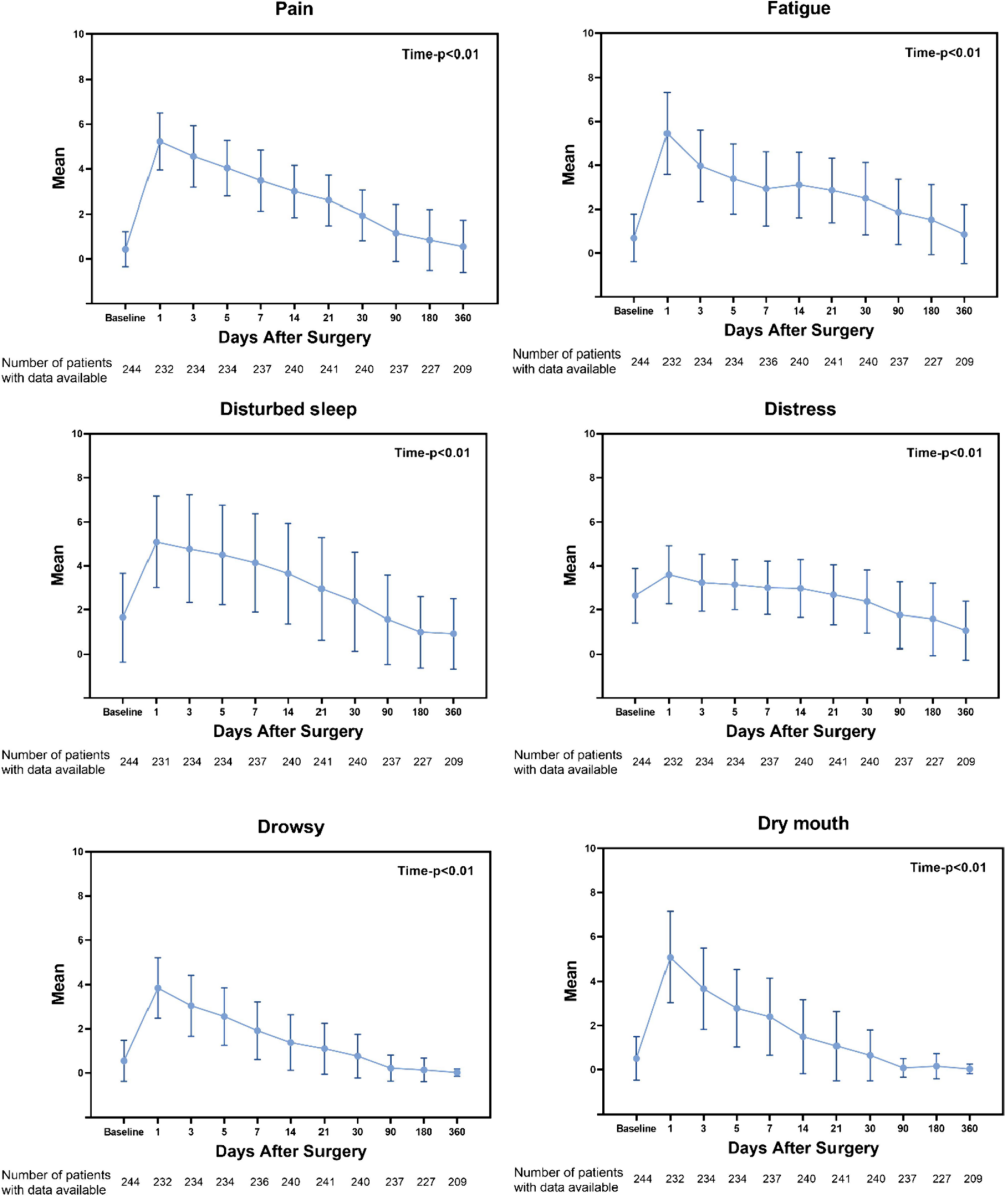
Patient-reported outcomes of symptoms after minimally invasive McKeown esophagectomy. Error bars represent standard errors. Linear mixed-effect models were used to evaluate whether symptoms significantly differed over time

**Fig. 4:**
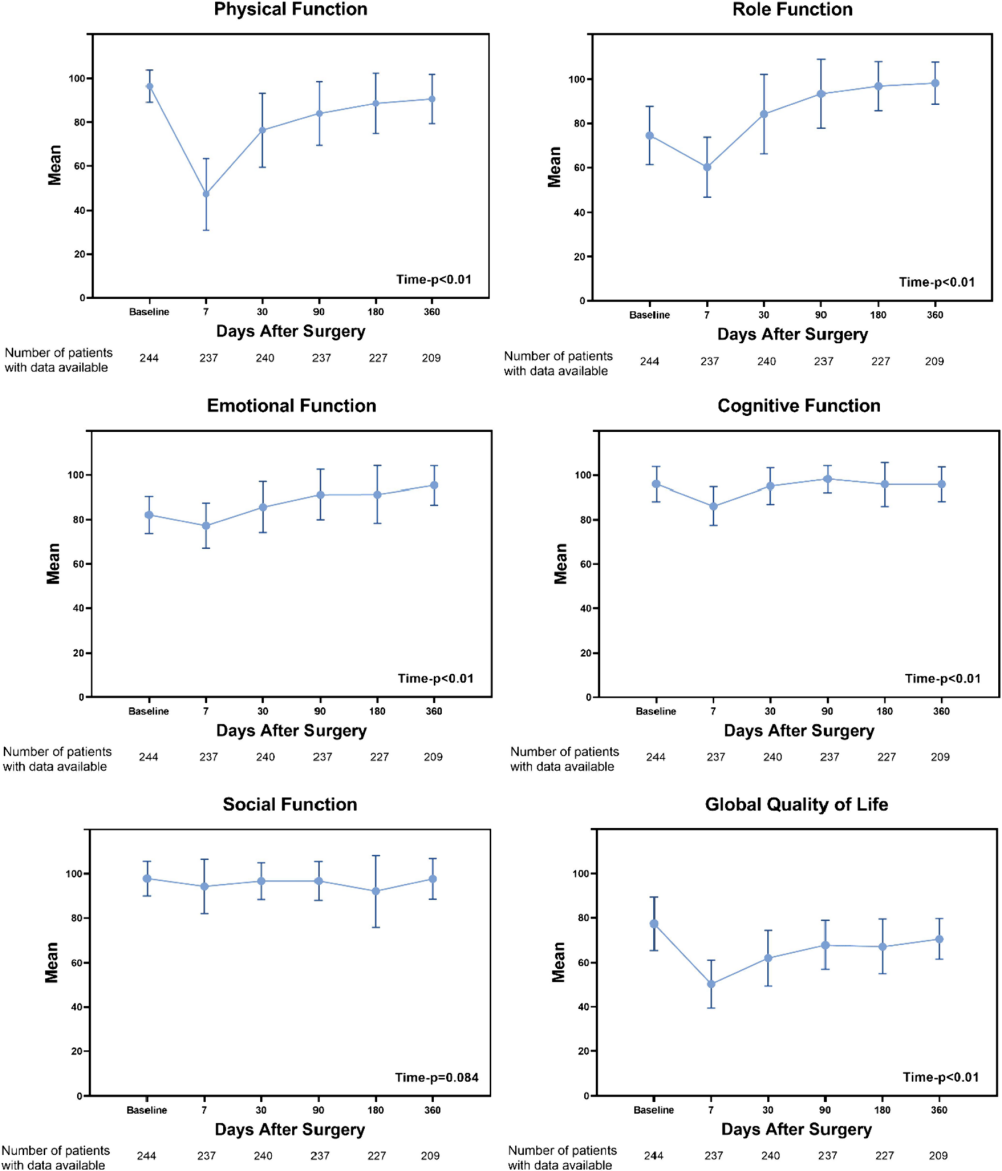
Patient-reported outcomes of quality of life after minimally invasive McKeown esophagectomy. Error bars represent standard errors. Linear mixed-effect models were used to evaluate whether quality of life significantly differed over time

Longitudinal changes in symptoms and QOL are shown in Figs. 3 and 4. Social functions did not show significant changes before and after surgery (*P* = 0.084). Physical function and global QOL did not return to the baseline level at 1 year after surgery, role function and emotional function returned to the baseline level at 1 month after surgery, and cognitive function returned to the baseline level at 3 months after surgery. The differences in pain, fatigue, disturbed sleep, distress, drowsiness, and dry mouth at each time point were statistically significant (*P* < 0.01). Pain and fatigue did not return to baseline levels at 1 year after surgery. Sleep restlessness, distress, drowsiness, and dry mouth returned to baseline levels within 3 months after surgery.

## Discussion

This study revealed trends in postoperative symptoms and QOL of patients with ESCC and their correlations. Notably, most symptoms had an incidence greater than 80% within 14 days after surgery. Different symptoms and QOL have different changes and recovery trends, and postoperative symptoms are important factors affecting patients’ QOL. In previous studies, traditional objective indicators were used to observe the QOL of patients with ESCC, often neglecting the patients’ own experiences. Applying PROs to clinical practice is an important aspect of perioperative care for patients with ESCC after surgery. Most studies of EC focused on endpoints such as postoperative survival and complications, often neglecting the QOL of patients after surgery. QOL is also a meaningful outcome for patients. Research has proven that the burden of postoperative symptoms persists, and poor QOL is also an important factor affecting the survival of patients [25].

A previous study showed that the QOL of patients with EC decreased at 1 to 3 months after surgery; then, QOL steadily increased, exceeding the baseline at 6 months and reaching the general population value 3 years later [26]. Another study also showed that for most patients who survive for 5 years after esophagectomy, QOL can recover to a level comparable to that of the general population; however, for patients with worsening conditions, QOL is difficult to recover and may even significantly decrease [11]. Those results were not exactly the same as our results. We analyzed the patients’ PROs within 1 year after surgery and collected PROs regarding symptoms and QOL seven times and two times, respectively, within 1 month after surgery; therefore, the results were more detailed and reflected the trend of changes in the patients’ symptoms and QOL during the short postoperative period (critical period of postoperative recovery). Patients have the heaviest burden of symptoms and the worst QOL within 7 days after surgery, but recovery trends were observed within 1 month. In the present study, the main symptoms of patients with ESCC were negatively correlated with the physical, role, emotional, cognitive, and social functions and global QOL. In other words, the heavier the patient’s symptom burden, the worse the QOL. Distress is one of the most important factors affecting patients’ QOL, especially 1 month to 1 year after surgery. It had the most apparent effect on emotional function (all *R*-values were > 0.71). At the same time, this study also analyzed the postoperative symptoms and QOL recovery process. Except for distress, which returns to the baseline level within 1 month, it requires time (at least 3 months) for most symptoms to return to the baseline level. At the same time, pain and fatigue are the most persistent symptoms and do not return to baseline at 1 year after surgery. Compared with other symptom outcomes in this study, postoperative pain and fatigue had the same recovery pattern but lasted longer and recovered more slowly. With a reduction in symptom burden, most patients’ QOL returned to baseline within 1 year. However, physical function and global QOL did not return to baseline levels 1 year after surgery, possibly because patients still had a particular symptom burden.

After surgery, patients with ESCC have a heavier burden of symptoms during the short term, which, to some extent, is related to the surgical reconstruction of the upper digestive tract required for the resection of EC after surgery [10]. Because of changes in lifestyle and diet, the psychological symptoms of patients with EC after surgery are severe, and anxiety and depression levels continue to increase after surgery. Furthermore, the operative time of esophagectomy is long, and the trauma is significant. Most patients with ESCC are older [27]. Additionally, QOL levels are lower for patients with ESCC than for patients with other gastrointestinal cancers [28]. These factors increase the symptom burden to a certain extent, thereby affecting patients’ QOL.

There are some notable points in this study. First of all, 76 patients had an ECOG score of 2. These patients were ambulatory and capable of self-care but unable to carry out work activities, and they were up and about > 50% of waking hours. Two patients had an ECOG score of 3, indicating they were capable of limited self-care and confined to bed/chair for > 50% of waking hours. After a thorough assessment of their physical condition (including lung function test, echocardiogram, dynamic electrocardiogram, and coronary computed tomography angiography) by surgeons and anesthesiologists, ensure that they have no surgical contraindications, and the surgery was decided based on multidisciplinary team discussions and joint discussions with patients. Another point is that 29% of patients received adjuvant therapy, including 12 cases that received neoadjuvant and adjuvant therapy and 56 cases that only received adjuvant therapy. The indications for adjuvant therapy mainly consider (1) high-risk factors such as non-pathological complete response, poorly differentiation, and vascular infiltration after neoadjuvant therapy combined with surgical treatment [29]; (2) pathological staging after direct surgery suggests high-risk factors such as multiple lymph node and multi-station metastases, poorly differentiation, and vascular infiltration; these decisions regarding adjuvant therapy are primarily based on multiple disciplinary team discussions and joint discussions with patients. Besides, among the 56 patients who underwent surgery without neoadjuvant therapy, there were 16 and 4 patients with clinical staging of stage III and IV, respectively. The main reason why these patients did not receive neoadjuvant therapy is determined by their circumstances, such as their financial situation and advanced age.

The main strength of this study was its prospective, population-based longitudinal design, which offset selection and recall biases. Complete follow-up was conducted with a large sample size and well-validated questionnaire that included preoperative symptom and HRQL assessments. The high response rate throughout the process ensured high statistical validity, and a clinically significant and reliable conclusion was obtained. However, this study had some limitations. First, the patient’s assessment of preoperative HRQL may be influenced by the cancer diagnosis, neoadjuvant therapy, and upcoming surgery. However, most patients recover their HRQL after neoadjuvant treatment before surgery [30, 31]. Second, the patient’s adjustment to the disease, shift in response, optimism level, and need for signs of clinical improvement throughout the treatment process may cause the assessment of symptoms to be biased [32, 33]. However, this response shift effect was similar across patients. Among 67.6% of patients in this study who did not receive neoadjuvant therapy, 26.1% of patients with pTNM stage I may have had better QOL because they did not receive chemotherapy and/or radiation before or after surgery, thereby increasing the average level.

In conclusion, the symptom burden of patients with ESCC after minimally invasive McKeown esophagectomy is obvious, peaks within a short time, and then continuously recovers towards the baseline level, and QOL improves as the burden of symptoms decreases. This study indicates that long-term HRQL after esophagectomy is acceptable. These findings have significant value for patients considering surgical and nonsurgical treatment during the preoperative phase, and they are of great significance for tracking patient recovery and implementing quality improvement measures.

## Data Availability

Data and analytical methods are available from the corresponding author upon reasonable request.

## References

[1] Bray F, Ferlay J, Soerjomataram I, Siegel RL, Torre LA, Jemal A (2018). Global cancer statistics 2018: GLOBOCAN estimates of incidence and mortality worldwide for 36 cancers in 185 countries. CA Cancer J Clin.

[2] Siegel RL, Miller KD, Wagle NS, Jemal A (2023). Cancer statistics. CA Cancer J Clin.

[3] Fitzmaurice C, Dicker D, Global Burden of Disease Cancer Collaboration (2015). The global burden of cancer 2013. JAMA Oncol.

[4] Liang H, Fan JH, Qiao YL (2017). Epidemiology, etiology, and prevention of esophageal squamous cell carcinoma in China. Cancer Biol Med.

[5] Obermannová R, Alsina M, Cervantes A (2022). Oesophageal cancer: ESMO Clinical Practice Guideline for diagnosis, treatment and follow-up. Ann Oncol.

[6] Gebski V, Burmeister B, Smithers BM, Foo K, Zalcberg J, Simes J (2007). Survival benefits from neoadjuvant chemoradiotherapy or chemotherapy in oesophageal carcinoma: a meta-analysis. Lancet Oncol.

[7] Viklund P, Lindblad M, Lu M, Ye W, Johansson J, Lagergren J (2006). Risk factors for complications after esophageal cancer resection: a prospective population-based study in Sweden. Ann Surg.

[8] Rutegård M, Charonis K, Lu Y, Lagergren P, Lagergren J, Rouvelas I (2012). Population-based esophageal cancer survival after resection without neoadjuvant therapy: an update. Surg.

[9] Jacobs M, Macefield RC, Elbers RG (2014). Meta-analysis shows clinically relevant and long-lasting deterioration in health-related quality of life after esophageal cancer surgery. Qual Life Res.

[10] Schandl A, Lagergren J, Johar A, Lagergren P (2016). Health-related quality of life 10 years after oesophageal cancer surgery*.* Eur J Cancer. Oxf Engl.

[11] Derogar M, Lagergren P (2012). Health-related quality of life among 5-year survivors of esophageal cancer surgery: a prospective population-based study. J Clin Oncol.

[12] Blazeby JM, Farndon JR, Donovan J, Alderson D (2000). A prospective longitudinal study examining the quality of life of patients with esophageal carcinoma. Cancer.

[13] Djärv T, Lagergren J, Blazeby JM, Lagergren P (2008). Long-term health-related quality of life following surgery for oesophageal cancer. Br J Surg.

[14] Biere SSAY, van Berge Henegouwen MI, Maas KW (2012). Minimally invasive *versus* open oesophagectomy for patients with oesophageal cancer: a multicentre, open-label, randomised controlled trial. Lancet.

[15] Mehran R, Baber U, Dangas G (2018). Guidelines for patient-reported outcomes in clinical trial protocols. JAMA.

[16] Basch E, Jia X, Heller G (2009). Adverse symptom event reporting by patients *vs* clinicians: relationships with clinical outcomes. J Natl Cancer Inst.

[17] Rotenstein LS, Huckman RS, Wagle NW (2017). Making patients and doctors happier – the potential of patient-reported outcomes. N Engl J Med.

[18] Blazeby JM, Kavadas V, Vickery CW, Greenwood R, Berrisford RG, Alderson D (2005). A prospective comparison of quality of life measures for patients with esophageal cancer. Qual Life Res.

[19] Cleeland CS, Mendoza TR, Wang XS (2000). Assessing symptom distress in cancer patients: the M. D Anderson Symptom Invent Cancer.

[20] Wang XS, Wang Y, Guo H, Mendoza TR, Hao XS, Cleeland CS (2004) Chinese version of the M. D. Anderson Symptom Inventory: validation and application of symptom measurement in cancer patients*.* Cancer, Chinese version 101(8):1890–1901. 10.1002/cncr.20448 10.1002/cncr.20448 15386315

[21] Bacorro WR, SyOrtin TT, Suarez CG (2015). Validation of the MD Anderson Symptom Inventory-Head-and-Neck-Filipino (MDASI-HN-F): clinical utility of symptom screening among patients with head-and-neck cancer. BMJ Support Palliat Care.

[22] Wang Y, Xie Z, Liu Y (2022). Symptom clusters and impact on quality of life in esophageal cancer patients. Health Qual Life Outcomes.

[23] Aaronson NK, Ahmedzai S, Bergman B (1993). The European Organization for Research and Treatment of Cancer QLQ-C30: a quality-of-life instrument for use in international clinical trials in oncology. J Natl Cancer Inst.

[24] Dai Z, Lang W, Yang H (2017). Validation of EORTC QLQ-OES18 for Chinese patients with esophageal cancer. Dis Esophagus.

[25] Wikman A, Johar A, Lagergren P (2014). Presence of symptom clusters in surgically treated patients with esophageal cancer: implications for survival. Cancer.

[26] Katz A, Nevo Y, Ramírez García Luna JL (2023). Long-term quality of life after esophagectomy for esophageal cancer. Ann Thorac Surg.

[27] Vlacich G, Samson PP, Perkins SM (2017). Treatment utilization and outcomes in elderly patients with locally advanced esophageal carcinoma: a review of the National Cancer Database. Cancer Med.

[28] Hart TL, Charles ST, Gunaratne M (2018). Symptom severity and quality of life among long-term colorectal cancer survivors compared with matched control subjects: a population-based study. Dis Colon Rectum.

[29] Lee Y, Samarasinghe Y, Lee MH (2022). Role of adjuvant therapy in esophageal cancer patients after neoadjuvant therapy and esophagectomy: a systematic review and meta-analysis. Ann Surg.

[30] van Meerten E, van der Gaast A, Looman CW, Tilanus HW, Muller K, Essink-Bot ML (2008). Quality of life during neoadjuvant treatment and after surgery for resectable esophageal carcinoma. Int J Radiat Oncol Biol Phys.

[31] Blazeby JM, Sanford E, Falk SJ, Alderson D, Donovan JL (2005). Health-related quality of life during neoadjuvant treatment and surgery for localized esophageal carcinoma. Cancer.

[32] Cella D, Hahn EA, Dineen K (2002). Meaningful change in cancer-specific quality of life scores: differences between improvement and worsening. Qual Life Res.

[33] Sprangers MA, Schwartz CE (1999). Integrating response shift into health-related quality of life research: a theoretical model. Soc Sci Med.

